# Policy lessons from comparing mortality from two global forces: international terrorism and tobacco

**DOI:** 10.1186/1744-8603-1-18

**Published:** 2005-12-15

**Authors:** George Thomson, Nick Wilson

**Affiliations:** 1 Department of Public Health, Wellington School of Medicine and Health Sciences, University of Otago, PO Box 7343, Wellington South, New Zealand

## Abstract

**Background:**

The aim of this study was to compare the mortality burdens from two global impacts on mortality: international terrorism and the major cause of preventable death in developed countries – tobacco use. We also sought to examine the similarities and differences between these two causes of mortality so as to better inform the policy responses directed at prevention.

**Methods:**

Data on deaths from international terrorism were obtained from a US State Department database for 1994–2003. Estimates for tobacco-attributable deaths were based on Peto et al 2003. The countries were 37 developed and East European countries.

**Results and discussion:**

The collective annualized mortality burden from tobacco was approximately 5700 times that of international terrorism. The ratio of annual tobacco to international terrorism deaths was lowest for the United States at 1700 times, followed by Russia at 12,900 times. The tobacco death burden in all these countries was equivalent to the impact of an 11 September type terrorist attack every 14 hours.

Different perceptions of risk may contribute to the relative lack of a policy response to tobacco mortality, despite its relatively greater scale. The lack is also despite tobacco control having a stronger evidence base for the prevention measures used.

**Conclusion:**

This comparison highlights the way risk perception may determine different policy responses to global forces causing mortality. Nevertheless, the large mortality differential between international terrorism and tobacco use has policy implications for informing the rational use of resources to prevent premature death.

## Background

International terrorism, or aspects of it, have been argued to be a reaction to globalization and/or to be aided by many of its features [1,2]. In the last twenty or more years, there has been a substantial focus on terrorism-related policies in many jurisdictions, particularly since the attacks of 11 September 2001 in the United States. This focus has included spending and legislation, and has included public health measures relating to bioterrorism protection [3,4]. The focus is understandable, considering the political significance of attacks by non-state organisations, and the economic and psychological effects on the societies which may consider themselves attacked [5-7]. However, it is important for policy makers to know of the opportunity costs of the response to international terrorism, relative to addressing other causes of premature death, and to better understand how differences in risk perception influence policy making. Therefore, we contrasted the mortality impacts of international terrorism with another major cause of preventable death – tobacco use [8] (which is also exacerbated by globalization [9,10]). This work is part of a wider attempt to put international terrorism into a public health context [11,12].

## Methods

As part of a study to describe the epidemiology of international terrorism [11] we extracted data for 1994–2003 on international terrorist attacks involving any deaths among non-perpetrators from United States (US) Department of State reports. The definition of terrorism used by the Department is: 'Premeditated, politically motivated violence perpetrated against noncombatant targets by subnational groups or clandestine agents', with international terrorism meant as 'terrorism involving citizens or the territory of more than one country'. These data were supplemented with findings from more detailed published studies (see: [11]). Countries included were 21 'established market economy' countries and 16 'former socialist economies of Europe' (as per the classification in an international mortality study) [13]. These two groups of countries were selected because there was better quality data available for both terrorism and tobacco. From these data, an average annual mortality burden was calculated for each country.

Data on tobacco mortality was based on the updated estimates for the year 2000 by Peto et al [14]. This method involves country-specific rates of lung cancer mortality together with corresponding rates from the American Cancer Society's Cancer Prevention Study II to derive 'smoking impact ratios' by age and sex. The burden includes tobacco-related: respiratory diseases, vascular diseases and other tobacco-related cancers. This methodology has been shown to be a robust indicator of the accumulated hazards of smoking [15].

Rates were calculated using the most recent population data for each country from the World Health Organization website .

## Results

For the selected countries collectively, the annual mortality burden from tobacco was approximately 5700 times that of the average annual mortality burden from international terrorism (Table 1). For 26 of the countries, there were no deaths from international terrorism. Within the other 11 countries, the ratio of annual tobacco to international terrorism deaths was lowest for the US at 1700 times, followed by Russia at 12,900 times.

**Table 1:** Mortality burdens from tobacco and international terrorism in developed and East European countries

**Country**	**Number of international terrorist attacks (1994–2003)**	Number of international terrorism deaths (1994–2003)**	**International terrorism deaths (per million population per year) (1994–2003)**	**Total deaths attributed to tobacco in year 2000 (estimated by Peto et al)**	**Ratio of tobacco deaths to annualized international terrorism deaths**
**Established market economies**					
Australia	0	0	0	18,600	-
Austria	0	0	0	8,900	-
Belgium	0	0	0	18,600	-
Canada	0	0	0	44,800	-
Denmark	0	0	0	11,600	-
Finland	0	0	0	5,200	-
France	7	19	0.032	64,900	34,158
Germany	2	3	0.004	111,100	370,333
Greece	3	3	0.027	13,400	44,667
Ireland	0	0	0	5,700	-
Italy	0	0	0	80,700	-
Japan	0	0	0	114,200	-
Netherlands	1	6	0.037	25,700	42,833
New Zealand	0	0	0	4,500	-
Norway	0	0	0	5,600	-
Portugal	0	0	0	8,400	-
Spain	1	1	0.002	46,400	464,000
Sweden	0	0	0	8,200	-
Switzerland	1	1	0.014	6,800	68,000
United Kingdom	3	32	0.054	114,000	35,625
United States	2	2,970	1.020	514,000	1,731

**Subtotal**	**20**	**3,035**	**0.351**	**1,231,300**	**4,057**

Former socialist economies of Europe*					
Belarus	0	0	0	18,000	-
Bulgaria	0	0	0	11,200	-
Croatia	0	0	0	8,000	-
Czech Republic	0	0	0	17,900	-
Estonia	0	0	0	2,700	-
Hungary	0	0	0	28,700	-
Latvia	0	0	0	4,100	-
Lithuania	0	0	0	4,700	-
Macedonia	0	0	0	2,000	-
Poland	1	1	0.003	68,700	687,000
Romania	0	0	0	31,900	-
Russia	7	256	0.178	330,000	12,891
Serbia & Montenegro	3	6	0.057	17,800	29,667
Slovakia	0	0	0	8,100	-
Slovenia	0	0	0	2,900	-
Ukraine	0	0	0	99,100	-

**Subtotal**	**11**	**263**	**0.081**	**655,800**	**24,935**

**Total (all selected countries)**	**31**	**3,298**	**0.278**	**1,887,100**	**5,722**

* Excluding Albania, and Bosnia and Herzegovina for which tobacco-related mortality burdens were not available. Moldova was not included in the WHO report from which this grouping comes.

** Excluding the deaths of perpetrators.

The absolute annual burden from tobacco was highest for the US at 514,000 deaths per year in 2000 (Table 1). This is equivalent to the impact of an 11 September type terrorist attack every 2.1 days. For all of these 37 countries collectively, the tobacco mortality burden was equivalent to the impact of an 11 September type terrorist attack every 14 hours.

## Discussion

Definitions of terrorism are highly contended [16-18]. Furthermore, we have identified some limitations with the US State Department dataset, including with the definition used [11]. Indeed, if a tighter definition of international terrorism was used, then this would substantially reduce the number of deaths categorised in this way (eg, relative to domestic terrorism or other types of homicide [11]). Therefore this analysis may over-represent the mortality burden from international terrorism to some degree.

In contrast, the tobacco mortality estimates may be underestimates of the true mortality burden. This is because the estimates by Peto et al ignore all deaths in those aged under 35 years (including neonatal deaths and deaths from sudden infant death syndrome attributable to smoking), and the methodology was one of 'conservative underestimation of tobacco hazards' [19]. More recent data also suggests that the long-term hazards of smoking on health are probably higher than previously thought [20]. Nevertheless, methodologies for assessing the tobacco-related mortality burden differ and for the US a more recent analysis [21] indicates a lower mortality burden attributable to tobacco (ie, 438,000 versus the 514,000 calculated by Peto et al and used in this analysis).

Despite these various limitations, the findings of this analysis suggest that the mortality burden from tobacco use is at present vastly greater than from international terrorism in all the selected countries studied. This is even the case for the US, which has suffered the worst mortality burden from international terrorism out of these countries in the last decade.

Why does tobacco mortality not receive a proportionate response? Some may find comparisons between 'catastrophic' and 'normal' deaths misplaced [22]. We recognise the subjectivity of risk perception [23,24], and the tendencies of populations to: (i) overestimate risks stemming from visible, well publicised sudden violence with collective results, particularly where the cause is not well understood, compared to risks with results dispersed over place and time; and (ii) to overestimate risks from causes were there is little apparent control by the individual, compared to risks from causes which appear to many to be voluntarily undertaken [25-27].

This tendency may be exacerbated by disproportionate media coverage of certain causes of mortality which involve low risk at the individual level [28,29]. There is also the political problem of giving priority to long-term issues, compared to dealing with emotive immediate concerns [30,31]. However, we have also demonstrated elsewhere that even for another cause of mortality which results in visible, well publicised sudden death (road crashes), policymaking does not appear to take into account the disproportionate mortality burden, compared to that from international terrorism [12].

International terrorism and the harm from tobacco use have similarities, in that they both involve discrete perpetrators – international terrorist groups and the globalized tobacco industry – against which governments can take action. Also, many tobacco deaths globally are due to the actions of foreigners – policymakers and company officials in tobacco manufacturing and exporting countries. Both international terrorism and tobacco use can substantially harm national economies and the international economic fabric in many ways [32,33]. Similarly, both can have widespread impacts on the way society functions and on its institutions eg, terrorism on security arrangements, and tobacco via the tobacco industry on the functioning of political processes [34,35]. The costs from both are largely or totally preventable, and investment in long-term prevention for both, as opposed to containment, may not necessarily be mutually exclusive (eg, if military budgets are diverted to terrorism prevention).

Despite these similarities, there are substantive differences. One is that the tobacco industry, unlike terrorists, is generally described as 'a legal industry' ie, an industry taking part in legal activity. This is despite the fact that the deliberate sale of a highly addictive, commonly lethal substance, and the routine denial of some harms (eg, of secondhand smoke) may be considered reckless criminal behaviour under the laws of some countries [36]. This presumed 'legality' contributes to the societal acceptance and political strength of the tobacco industry in developed countries, relative to international terrorist groups.

Secondly, there is considerable evidence about the preventability of tobacco-related harm using current methods, and of their cost-effectiveness [37-40], compared to the high uncertainty about the effectiveness of particular measures to prevent international terrorism or its health impacts [41,42]. From a public health perspective, anti-terrorism efforts tend to focus on immediate containment, rather than addressing the possible root causes of terrorism [43-46]. The cost-effectiveness of public health measures related to potential terrorism impacts has had little conclusive research [47,48].

A further difference, as this analysis indicates, is the vastly different *scale *of the consequent mortality burdens. The policy implications of this include the relative extent, effectiveness and cost-effectiveness of the resources used to address the two problems [49-52]. A public health and evidence-based approach may suggest a greater relative emphasis on tobacco control both nationally and internationally. While public health budgeting will always have to take into account public concerns that are not based on the evidence of relative risks, we argue that such policy moves should be as rigorously examined, as is the budgeting for tobacco control. A further possible implication is to learn from the response to international terrorism, so as to inform the way that tobacco marketing can be reframed as a serious threat to the social and economic well-being of individual countries and to international social and economic development.

## Conclusion

This analysis suggests a very large mortality differential between these two problems exacerbated by globalization, international terrorism and tobacco use. Different perceptions of risk may contribute to the relative lack of a policy response to tobacco mortality, despite its greater scale. The lack of an appropriate response is also despite tobacco control having a stronger evidence base for the prevention measures used. National and international policy makers need to consider these issues if they are to make more rational use of resources to prevent premature mortality.

## Competing interests

Both authors have undertaken contract work for tobacco control-related non-government agencies, and NW has undertaken contract work in tobacco control for the New Zealand Ministry of Health.

## Authors' contributions

Both authors contributed to the design of the study, the data collection and the drafting and final write up of the manuscript. NW undertook the data analysis.
